# Integrating health into the European Union Migrant and Asylum Pact: a call to action

**DOI:** 10.1016/j.lanepe.2024.101096

**Published:** 2024-10-09

**Authors:** Angelo Farina, Doris Zjalic, Federico Fama, Sara Laura Ferrari, Andrea Gori, Mario Raviglione

**Affiliations:** a Center for Global Health Research and Studies UCSC, Department of Life Sciences and Public Health, Università Cattolica del Sacro Cuore, Rome, Italy; b Centre for Multidisciplinary Research in Health Science (MACH), University of Milan, Italy; c Infectious Disease Unit II, Ospedale Luigi Sacco, ASST Fatebenefratelli Sacco, Milan, Italy; d Department of Biomedical and Clinical Sciences, University of Milan, Italy

In December 2023, the Council of the European Union and the European Parliament reached an agreement on the new Migration and Asylum Pact, a set of new rules that reforms migration policies in the EU.^1^ This Pact is made up of four pillars: secure external borders, fast and efficient procedures, effective system of solidarity and responsibility, and embedding migration in international partnerships. The number of new migrant arrivals in Europe has fluctuated in recent years, driven by factors like conflict, economic instability, and climate change. As of 2024, Europe continues to face challenges in managing migration flows, with a mix of humanitarian needs and political tensions shaping responses. Looking ahead, the situation is likely to remain complex, with ongoing pressures from regions like Africa and the Middle East. Climate change and geopolitical instability may exacerbate these trends, potentially leading to increased migration.^2^

The epidemiological situation of migrants arriving in Europe must be considered as it has different implications: the early diagnosis and correct treatment related to the human right to health, and the correct management of communicable and non-communicable diseases relates to public health and important economic implications. On arrival, refugees are particularly vulnerable to infectious diseases due to the collapse of healthcare systems in their home countries, including disrupted vaccination services and public health infrastructure, but also to accidental injuries, hypothermia, gynecological and obstetric complications, gastrointestinal and respiratory illnesses, dermatological, cardiovascular events, mental illness and metabolic problems.^3^

Migrants have distinct health profiles compared to local-born residents, adding diversity to Europe's health landscape, especially in non-communicable diseases (NCDs).^3^ Evidence shows a higher diabetes mortality among migrants from regions like North Africa, the Caribbean, and South Asia.^4^ West African migrants face higher rates of hypertension and stroke, while those from Afghanistan and Northern Africa are more prone to coronary heart disease.^5^^,^^6^ Migrants are also more susceptible to early-life infection-related cancers, such as liver and cervical cancer, but less likely to develop cancers linked to a Western lifestyle, like colorectal and breast cancer.^3^ This picture explains the importance of taking into account the migrants' health specificities when organizing preventive programmes (e.g. vaccination, cancer screening, infectious diseases screening) and health assistance. Despite the evident necessity for tailored healthcare provisions for migrants, the implementation of EU directives, such as the 2013/33/UE^7^ which outlines the assessment of special health needs, has remained inadequate. Subsequently there has been a notable absence of legislative developments concerning the protection of the migrants’ health.

Nowadays the EU does not have common and consistent health policies. On the topic of migrant screening, as proven in the latest agreement, only identification and fingerprinting have been ratified. The Council of the European Union did not worry about the health of the migrants upon arrival, a mandatory human right but also an important act of public health management for its own intern citizenship. This regulatory deficit, exacerbated by the failure to implement common health policies in the new Pact of Migration and Asylum contributes to the absence of a coherent approach to the health of migrants and asylum seekers.

Based on these growing data, it is clear that an emergency approach to health, not even amended by the current Pact, is unsuitable. Engagement from the European scientific community is imperative to ensure a path for access to care and to optimize the cost-effectiveness of interventions. To rapidly improve the situation, there is the need for of crucial interventions (Panel 1).

Panel 1 **Interventions suggested by the authors to a holistic approach to migrant care.**

**Table:** 

1. Creation of evidence-based policies backed by adequate human, structural, and financial resources from European Union Agency for Asylum, EUAA,^7^ encompassing comprehensive interventions that include the provision of first aid, availability of well-equipped health services with a wide-range screening capacity, and special attention to infectious diseases;
2. Establishment of effective EU-wide referral systems taking into account rapid redistributions of migrants to final destinations, but prioritizing health issues, a function now partially but not fully covered by EUAA^8^;
3. Production of clear and realistic technical guidelines drawn up by a multidisciplinary and cross-sectoral pool of specialists and adapted to local conditions, through a process of collaboration between universities, European and local institutions;
4. Development of a standard digital system for data collection and sharing among the different health facilities involved in migrants’ care and useable for continuous monitoring of guideline application, through the development of user-friendly KPIs by European agencies such as eu-LISA^9^;
5. Training and empowerment of healthcare workers to promote ideal care standards among migrants and contribute to academic and political discussions with key stakeholders;
6. Intensified educational efforts to improve healthcare for migrants with the inclusion of migrant health as a discipline in medical curricula and promotion of public health priorities in schools, arts and society, through the contribution of EACEA - European Education and Culture Executive Agency.^10^

Health should be at the core of any discussion about migration as a fundamental human right enshrined in the WHO constitution and that of each European country. Substantive, evidence-based public engagement in the discourse should be raised as a priority issue within the European borders. Seeking a clear commitment to health as a crucial aspect of migration policy is not only in accordance with international legal frameworks but also enhances the importance of evidence-based decision-making in shaping public policies.

## Contributors

Conceptualization: AF; Writing, original draft: DZ; Writing, review and editing: AF, DZ, FF, SLF, AG, MR; Supervision: AG, MR. All authors read and approved the final manuscript.

## Declaration of interests

None declared.
